# Visual system plasticity in mammals: the story of monocular enucleation-induced vision loss

**DOI:** 10.3389/fnsys.2015.00060

**Published:** 2015-04-28

**Authors:** Julie Nys, Isabelle Scheyltjens, Lutgarde Arckens

**Affiliations:** Laboratory of Neuroplasticity and Neuroproteomics, KU LeuvenLeuven, Belgium

**Keywords:** deafferentation, age, deprivation, multimodal, reorganization, cortical plasticity, visual system

## Abstract

The groundbreaking work of Hubel and Wiesel in the 1960’s on ocular dominance plasticity instigated many studies of the visual system of mammals, enriching our understanding of how the development of its structure and function depends on high quality visual input through both eyes. These studies have mainly employed lid suturing, dark rearing and eye patching applied to different species to reduce or impair visual input, and have created extensive knowledge on binocular vision. However, not all aspects and types of plasticity in the visual cortex have been covered in full detail. In that regard, a more drastic deprivation method like enucleation, leading to complete vision loss appears useful as it has more widespread effects on the afferent visual pathway and even on non-visual brain regions. One-eyed vision due to monocular enucleation (ME) profoundly affects the contralateral retinorecipient subcortical and cortical structures thereby creating a powerful means to investigate cortical plasticity phenomena in which binocular competition has no vote.In this review, we will present current knowledge about the specific application of ME as an experimental tool to study visual and cross-modal brain plasticity and compare early postnatal stages up into adulthood. The structural and physiological consequences of this type of extensive sensory loss as documented and studied in several animal species and human patients will be discussed. We will summarize how ME studies have been instrumental to our current understanding of the differentiation of sensory systems and how the structure and function of cortical circuits in mammals are shaped in response to such an extensive alteration in experience. In conclusion, we will highlight future perspectives and the clinical relevance of adding ME to the list of more longstanding deprivation models in visual system research.

## Introduction

The capacity of the mammalian brain to rewire and physiologically modify neural connections in response to environmental changes is an intriguing and evolutionary conserved feature. Plastic modifications can have distinct causes and purposes but in general the brain operates in a state of ongoing plasticity (Pascual-Leone et al., 2005). Given the diversity of its functions, various types of plasticity including synaptic, homeostatic and structural plasticity, are present across distributed neural networks in both juveniles and adults. They typically operate in parallel to allow specific changes at the molecular, cellular, systems and behavioral level as well as to allow compensational or homeostatic changes at the network level (Turrigiano and Nelson, 2000; Citri and Malenka, 2008; Holtmaat and Svoboda, 2009).

When a sensory system fails, as in blindness or deafness, the remaining senses can recruit the “non-stimulated” brain areas by making new or by potentiating existing connections. At the same time, they can strengthen their own functional processing of sensory input to compensate for the loss of the other sense. This phenomenon is defined as cross-modal plasticity. Indeed, extensive reports in rodents and higher-order mammals like cat, monkey, and humans describe cross-modal plasticity in response to complete loss of a sensory modality early in life (for review see Bavelier and Neville, 2002; Merabet and Pascual-Leone, 2010). Nevertheless, accumulating evidence also supports the presence of such plasticity in adulthood and even after partial sensory deprivation (Newton et al., 2002; Allman et al., 2009a; Meredith et al., 2012; Maslin et al., 2013) substantiating the notion of the capacity for brain plasticity throughout life.

The visual system of many mammals has been extensively studied to unravel the basic working principles of neuronal physiology, development and plasticity. Reports on the visual cortex are numerous and it is one of the best-described brain areas in relation to the principle of structure-function coupling. Also, the clear dissimilarity of sensory response properties between cortical and subcortical brain regions allows the identification of exclusive cortical attributes (Espinosa and Stryker, 2012). Since experience acts as a potent force to shape neural circuits and ultimately behavior, the relatively straightforward manipulations of vision in different animal models continue to help decipher the driving forces behind experience-dependent neuronal plasticity. Popular experimental paradigms for the study of visual impairment range from invasive methods such as eyelid suture (mouse: Gordon and Stryker, 1996; Levelt and Hübener, 2012), splitting the optic chiasm (Berlucchi and Rizzolatti, 1968; Yinon and Hammer, 1985) and intraocular injection of tetrodotoxin (TTX; Frenkel and Bear, 2004) to non-invasive methods including dark rearing from birth (Morales et al., 2002; Gianfranceschi et al., 2003; Kreczko et al., 2009; Yang et al., 2013), dark exposure (shorter period with varying starting point: He et al., 2006, 2007; Huang et al., 2010; Montey and Quinlan, 2011; Guo et al., 2012; Duffy and Mitchell, 2013; Petrus et al., 2014), eye patching (Zapasnik and Burnat, 2013; Laskowska-Macios et al., 2014), stimulus-specific exposure like stripe (Blakemore and Cooper, 1970; Blasdel et al., 1977) or strobe rearing (Humphrey and Saul, 1998) and the application of prism goggles (Yoshitake et al., 2013). These studies have led to the characterization and timing of critical periods^1^ for ocular dominance and also for direction and orientation selectivity.

Monocular enucleation (ME) or the surgical removal of one eye can be considered a model for unilateral sensory deafferentation where half of the normal visual input is lost. In this type of drastic vision loss even low contrast vision, which still occurs through sutured eye lids, as well as any form of retinal spontaneous activity from the manipulated eye is lost. It has been used for the first time in neonatal rabbits as early as 1870 to trace the course and destination of eye-specific neuronal projections across the visual system (Gudden, 1870). These primordial observations together with subsequent studies in rats, cats and dogs consistently revealed histological alterations and marks of degeneration along the ascending retino-geniculate and—collicular pathway following ME early in life. Numerous studies in the following century collectively contributed to our current understanding of enucleation-induced subcortical structural alterations, including the extension of retinal afferents originating from the remaining eye in the lateral geniculate nucleus (LGN), and superior colliculus (SC) (for review see Toldi et al., 1996). Overall, they revealed the topographic maturation of distinct retinal projections to the contra- and ipsilateral target regions inside the LGN and SC. In comparison, the contralateral visual cortex appeared less prone to the anterograde degenerative mechanisms of ME (Tsang, 1937). Nevertheless, visual areas, especially ipsilateral to the remaining eye, displayed an enlarged distribution of callosal connections in adulthood such that they are no longer limited to the strip across the border between primary and extrastriate cortices (Wree et al., 1985; Olavarria et al., 1987). These observations are in relation to less extensive pruning of axons of callosal neurons due to the early lack of input from one eye.

Experience-dependent neuroplasticity occurs throughout life and as such has gained ample interest from the 1970’s onwards (Wall and Egger, 1971). Indeed, a vast body of literature has demonstrated that the mature neocortex is not a fixed entity but retains substantial malleability, which is exemplified in primates (Kaas et al., 1983; Kaas, 1991; Donoghue, 1995; Gilbert, 1998; Qi et al., 2014), cat (Chino et al., 1995; Darian-Smith and Gilbert, 1995; Hu et al., 2009, 2010, 2011), ferret (Erisir and Harris, 2003; Allman et al., 2009a), raccoon (He et al., 2004), rat (Siucinska and Kossut, 1994; Kossut, 1998; Zhou et al., 2011; Tandon et al., 2013) and mouse (Keck et al., 2008; Lehmann and Löwel, 2008; Maya-Vetencourt et al., 2008; Van Brussel et al., 2011). In this context ME applied later in life has been a valuable research model since it could reveal additional plasticity and physiological modifications in the mature sensory cortex as compared to the other invasive and non-invasive vision impairment models (Newton et al., 2002; Paulussen et al., 2011b; Van Brussel et al., 2011; Nys et al., 2014).

This review will mainly emphasize on the effects of ME within the visual system of mammals. Since age at the time of surgical intervention is a decisive factor for the subsequent alterations, including a different degree of plasticity in subcortical structures and sensory cortex, we will highlight how age next to the type of visual manipulation is paramount towards understanding the multifaceted aspects of developmental, visual and cross-modal plasticity. Observations in ME animal models and humans will be compared and contrasted with observations in the blind.

## Monocular Enucleation as a Tool to Map Eye-Input Specific Subdivisions Within Visual Cortex

The areal map for the mouse visual cortex, including the number and identity of different visual areas is still constantly being refined (Garrett et al., 2014). A decade ago, no clear consensus about the number of areas and their functional organization, including the specific location of monocular and binocular driven zones, was available. Historical efforts to adequately delineate the different visual areas entail neuroanatomical methods like (immuno)histology (Caviness, 1975; Van der Gucht et al., 2007; Paulussen et al., 2011a) and tracer-based mapping (Olavarria and Montero, 1989; Wang and Burkhalter, 2007) next to *in vivo* electrophysiological (Wagor et al., 1980; Wang and Burkhalter, 2007; Van den Bergh et al., 2010; Vreysen et al., 2012) and intrinsic optical imaging approaches (Schuett et al., 2002). In this context ME was specifically applied to characterize the eye-input specific subdivisions within the mouse visual cortex. Indeed, the analysis of visually driven molecular activity patterns in the brain of mice with one or two eyes enucleated based on the expression of activity reporter genes like *zif268* and *c*-*fos*,^2^ identified the full spatial extent of the visual cortex along the anterior-posterior and medio-lateral axes of the brain as well as the different monocular and binocular driven subregions therein (Van Brussel et al., 2009; Aerts et al., 2014a). In fact, the monocular and binocular zones derived from that study correspond well with the representations of the monocular and binocular visual field(s) in the topographic map of mouse visual cortex by Wang and Burkhalter (2007). Meanwhile, advanced analysis by intrinsic optical imaging (Kalatsky and Stryker, 2003; Garrett et al., 2014), two-photon calcium imaging methods (Andermann et al., 2011; Marshel et al., 2011; Roth et al., 2012; Glickfeld et al., 2013) and modern anatomical studies combined with network analysis (Wang et al., 2011, 2012; Wang and Burkhalter, 2013) made it possible to study the functional and connectional properties of V1 and each of the extrastriate areas in the mouse in more detail.

A major difference between the visual cortex of rodents and higher-order mammals is the salt-and-pepper configuration or “intermingled” organization of functionally linked neurons instead of the cortical columns of neurons with a similar ocular dominance, orientation and direction selectivity found in carnivores and primates (Dräger, 1975; Niell and Stryker, 2008; Gao et al., 2010). Actually, by virtue of the high percentage of crossing-over of the anatomical connections at the optic chiasm and the lack of ocular dominance columns in mouse and rat visual cortex, ME results in the irreversible loss of visual input to distinct contralateral monocular cortical target regions as elegantly shown in the mentioned ME investigations of rodent visual cortex structure (Toldi et al., 1996; Van der Gucht et al., 2007; Van Brussel et al., 2009).

In higher order mammals like primates and cats, this explains why ME can only result in complete loss of vision in those cortical regions that represent the monocular crescent of the peripheral visual field that is normally provided by the nasal retina of the removed eye (Eysel, 1979; Horton and Hocking, 1996). Monocular enucleation nevertheless revealed evolutionary differences in the functional ocular dominance columns in primary visual areas of distinct primate species. A comparative study based on the examination of cytochrome oxidase activity patterns after ME in adulthood revealed the absence of ocular dominance columns across layer III, IV and VI of V1 in the New World squirrel monkey in contrast to a clear eye-input specific organization in the Old World macaque monkey (Hendrickson and Tigges, 1985; Takahata et al., 2014). Other studies using intrinsic optical imaging of V1 confirmed the existence of ocular dominance columns in the New World owl monkey (Kaskan et al., 2007) and the Prosimian Bush baby (Xu et al., 2005). Using a similar approach of ME and cytochrome oxidase histochemistry in macaque monkeys, Horton and Hocking (1996) could demonstrate the presence of intrinsic variability in the periodicity of ocular dominance columns in layer IVc from animal to animal of the same species. In a subsequent study, they compared the effects of ME with eyelid suture and retinal laser lesions on cytochrome oxidase activity in the striate cortex of macaque monkeys. They revealed an additional functional parcellation of monocular core zones alternating with binocular border strips outside layer IVc in both monkey and human visual cortex (Horton and Hocking, 1998). Others have assessed neuronal activity in the Vervet monkey by means of expression analysis of activity reporter genes. Similar to cytochrome oxidase activity patterns, Zif268 and c-Fos immunoreactivity after monocular deprivation (lid suture, enucleation and TTX injections) revealed ocular dominance columns as well as their respective size and density (Chaudhuri et al., 1995, 1997; Van der Gucht et al., 2000).

## Monocular Enucleation as a Brain Plasticity Model

### Monocular Enucleation vs. Monocular Deprivation—Impact on Binocular V1

An important difference between ME and the frequently used MD paradigms, like lid suturing or eye patching, is that in case of ME, all retinal activity from one eye, including spontaneous waves and light-driven patterns, is instantly and irreversibly eliminated. Upon MD, the well-structured spontaneous retinal waves from one eye are replaced by uncorrelated noise and transferred via the LGN towards the visual cortex where it induces synaptic long-term depression (LTD; for review see Cooper and Bear, 2012). Upon ME no retinal input is left yet spontaneous synchronous bursting can still occur within the LGN, preventing cortical LTD and likely originating from cortico-thalamic feedback (Weliky and Katz, 1999). There is a complete removal of inhibitory binocular interactions following ME, which is responsible for the absence of binocular competition as a factor contributing to subsequent experience-dependent cortical plasticity (Hübener, 2003; Van Brussel et al., 2009). Consequently, the MD paradigms have been particularly instrumental in understanding the contribution of the correlation of binocular inputs as well as of high quality patterned vision in sculpting cortical circuits during development (Morales et al., 2002; Konur and Yuste, 2004; Burnat et al., 2012; Espinosa and Stryker, 2012; Zapasnik and Burnat, 2013; Chen et al., 2014). They will therefore remain the dominant methods of deprivation to understand ocular dominance and its plasticity, in relation to diseases like amblyopia (lazy eye) (Hofer et al., 2006; Morishita and Hensch, 2008; Levelt and Hübener, 2012; Sengpiel, 2013).

ME on the other hand allows to model other aspects of long-term vision loss, which are more difficult to accomplish and to maintain using for example pharmacological injections (i.e., TTX) in the eye. It is noteworthy that, in response to ME, histological alterations have been documented in subcortical vision centers (see Sections Effect of monocular enucleation at birth and Subcortical effects of monocular enucleation in adulthood), yet in the mouse visual cortex, only a negligible influence of injury artifacts of enucleation has been found. For instance, Smith and Trachtenberg (2007) demonstrated that pharmacological silencing of one eye without deafferentation of the optic nerve, results in a similar reduction of contralateral cortical activity as ME (Smith and Trachtenberg, 2007).

Ever since the pioneering studies of Cynader and colleagues (Shaw et al., 1986; Prasad and Cynader, 1994; Prasad et al., 2002), there has been an intense focus on identifying potential mechanisms that regulate plasticity and could control critical periods. In this context, gene expression profiling studies in both mouse (Majdan and Shatz, 2006) and old-World monkey (Lachance and Chaudhuri, 2004) did recognize ME as a proper and robust deprivation paradigm to elucidate candidate “plasticity genes” that are particularly sensitive to alterations in visual input during the traditional critical period of ocular dominance plasticity. In these studies, ME was exactly chosen because it induces robust changes in the eye-specific circuitry and it has been proven to globally change visual cortex gene expression (Chaudhuri et al., 1995). Although MD was shown to be useful in similar molecular studies (Rietman et al., 2012), gene expression changes turned out more reproducible upon ME than upon MD or monocular inactivation with TTX exactly because of variable levels of residual retinal activity in these visual deprivation paradigms (Majdan and Shatz, 2006).

The overall effect of MD and ME on binocular neurons in V1 is comparable, namely an ocular dominance shift towards the open eye (Faguet et al., 2009). Since ME induces the most robust intraocular activity imbalance possible, the signal to noise ratio in, for example, molecular activity mapping studies is maximal, in line with the above mentioned gene expression studies (Kanold et al., 2009; Van Brussel et al., 2009). Indeed, *arc* (activity-regulated cytoskeletal-associated protein) is one of the frequently used IEGs that can be specifically induced in V1 neurons by visual stimulation (Syken et al., 2006; Tagawa et al., 2005). When ME is performed in mice during the critical period (age P28), the *arc* expression in contralateral V1 in response to stimulation of the non-deprived open eye expands into closed-eye territory after a few days reflecting a spatial representation of the robust ME-induced ocular dominance plasticity (Tagawa et al., 2005; Syken et al., 2006; Datwani et al., 2009; Kanold et al., 2009).

Nevertheless in MD and ME different activity-dependent (synaptic and homeostatic) changes will likely occur in the contralateral visual cortex. For example, the amount of homosynaptic LTD of deprived connections, which is stronger when asynchronous (de-correlated) afferent activity is present, is probably less abundant upon ME. Hence, similar to monocular inactivation with TTX, ME will induce less LTD in the binocular visual cortex (Rittenhouse et al., 1999; Frenkel and Bear, 2004; Coleman et al., 2010). Furthermore, it is expected that activity-dependent modifications in both local and long-range intracortical connectivity patterns of GABAergic and pyramidal neurons, respectively (Trachtenberg et al., 2000; Calford et al., 2003; Erisir and Harris, 2003; Allman et al., 2009a; Keck et al., 2011; Vasconcelos et al., 2011), are differentially modulated upon ME and MD. Especially after long time periods, these two deprivation methods will likely cause a different recalibration of the excitation-inhibition balance, inside binocular V1, and certainly also in adjacent monocular cortical territories. Pronounced effects in the monocular cortex would not depend upon the mechanisms that underlie ocular dominance plasticity but rather implicate a broad contingent of distinct plasticity mechanisms, such as homeostatic synaptic scaling operating across the visual cortex after an altered regime of neural activity (Turrigiano et al., 1998; Goel et al., 2006; Mrsic-Flogel et al., 2007).

Summarized, complementary to MD studies in the critical period, different results in the ME model can reveal additional information regarding *deprivation-specific* mechanisms at play across visual areas whereas similar results between ME and MD could illustrate *general* mechanisms that take place after the loss of visual input, regardless of the severity of input removal.

### Effect of Monocular Enucleation at Birth

Enucleation of one eye at birth obviously interferes with the development of vision. Drastic structural rearrangements and changes in synaptic efficiency are induced along the subcortical, thalamocortical and cortico-cortical pathways, especially contralateral to the removed eye (for review see Toldi et al., 1996). In subcortical (Lund et al., 1973; Yagi et al., 2001; Chan et al., 2011; Furman and Crair, 2012) and cortical (Toldi et al., 1994b, 1996; Hada et al., 1999; Yagi et al., 2001) structures of the rodent visual system, the ME-induced rerouting of retinogeniculate, retinotectal and geniculocortical afferents and callosal inputs corresponds with the recruitment of deafferented neurons and the functional modifications in favor of the remaining eye. This enucleation-dependent reorganization of the uncrossed, ipsilateral visual pathway during development mirrors the perceptual learning ability of enucleated rats exposed to a black-white and horizontal-vertical discrimination task. Once the task has been learned, a lesion in the contralateral cortex, the ipsilateral cortex or the contralateral optic tract relative to the remaining eye was performed. An ipsilateral lesion resulted in retained learning skills in both the neonatal ME and late ME group whereas in the case of a contralateral lesion, only the neonatal ME rats were able to preserve memory (Yagi and Sakai, 1979; Sakai et al., 1991). In addition, visual acuity of the remaining eye in neonatally enucleated rats is significantly enhanced at 3 months of age (Sakai et al., 1996).

Remarkably, if a neonatal induced lesion in the visual cortex of kittens is combined with ME, the retrograde severe loss of X-type retinal ganglion cells, with high spatial resolution and low contrast thresholds and linked to the form-sensitive visual pathway, in the remaining eye is prevented. This retinal rescue suggests an ME-induced neuroprotection of retrograde cells normally degenerated by cortical damage (Illig et al., 1998). This is in line with the reduced apoptosis of retinal ganglion cells and preservation or even expansion of their connections in the remaining eye observed following early monocular vision (Guillery, 1989; Steeves et al., 2008).

Next to systems level changes, early ME in animals has uncovered certain molecular players involved in the development of afferent visual pathways. It is proposed that BDNF (and its TrkB receptor) levels are altered across retinotopic targets upon early ME. In this scenario, BDNF or other neurotrophic factors could initially decrease due to the loss of retinal input but, after long-term survival, are produced or secreted by different sets of local cells or delivered by anterograde or retrograde trafficking through neuronal pathways (Frost et al., 2001). Moreover, the transcription factor CREB (Vierci et al., 2013) and the matrix metalloproteinase 9 (Oliveira-Silva et al., 2007) have been implicated in the establishment and plasticity of retinotectal projections in rat and mouse upon ME.

The metabolic and biochemical mechanisms that accompany the ME-induced plasticity at early ages consist of changes in glucose utilization (Vargas et al., 2001; Wang et al., 2005) and neurotransmitter levels (Nakamura et al., 1984; Riback and Robertson, 1986). Recently, they have been evaluated *in vivo* using proton magnetic resonance spectroscopy of the visual cortex 3 weeks post-enucleation. The metabolic outcome likely reflects cortical reorganization associated with a general neural activity loss, the elimination of neurons and retraction of axon terminals (Chow et al., 2011).

The cytoarchitectonic structure of the visual cortex of neonatal ME mice, assessed by Golgi and histological methods, undergoes a reduction in the neuropil volume, an increase in neuronal densities, a higher variation in the dendrite orientation of stellate cells with ascending projections and a decrease in the number of dendritic spines of layer V pyramidal neurons (Valverde, 1968; Heumann and Rabinowicz, 1982). Furthermore, it appears that supragranular layers II and III of both contra- and ipsilateral visual cortex are most affected by neonatal enucleation (Heumann and Rabinowicz, 1982). In newborn ferrets that underwent ME the formation of orientation, spatial frequency and retinotopic maps is unaffected, but their structure and spatial relationships are altered compared with normal development in binocular intact animals (Farley et al., 2007).

### Subcortical Effects of Monocular Enucleation in Adulthood

At the cellular level, a glial response prevails across different subcortical direct retinal target structures in the adult mouse upon ME, as an early marker of neuronal injury (Cuyvers et al., 2010). Instant denervation-induced microglial activation precedes astrogliosis mainly in contra- but also in ipsilateral subcortical structures, including the LGN and SC (Wilms and Bähr, 1995; Gonzalez et al., 2006). In general, activated glial cells are known to clean up axonal debris, in this case of lost retinal ganglion cells, to restore tissue homeostasis and to release growth factors and cytokines to stimulate neuronal sprouting (Bechmann and Nitsch, 1997). Yet in adulthood, enucleation triggers a reduction of trophic influences in direct retinal targets in the brain. For example, BDNF levels in the LGN and SC of adult enucleated rats are significantly decreased (Avwenagha et al., 2006). Reactive oxygen species, which at non-toxic levels act as messenger molecules to mediate structural remodeling, are also apparent in subcortical structures of the adult rat visual system upon ME (Hernandes et al., 2010). Other manipulations at eye level, which involve retinal ganglion cell loss, and mimic visual disorders characterized by RGC death, also induce a glial response in the brain. For example, laser-induced monocular hypertension (mOHT), a mouse model for glaucoma, induces astrogliosis in the left and right SC and LGN of the mouse (Dekeyster et al., 2015), just as observed in primate OHT models (Lam et al., 2009; Shimazawa et al., 2012) and in optic nerve heads of human glaucomatous eyes (Prasanna et al., 2002) and this could correlate with the neurodegeneration and atrophy observed in the LGN of glaucoma patients (Gupta et al., 2006).

## Cross-Modal Plasticity: An Intriguing Response to Sensory Input Loss at Subcortical and Cortical Level

Cortical reorganization upon complete sensory deprivation does not only occur within the affected sensory system but is also present in other modalities. It is a popular belief that profound deprivation or denervation of one sense early in life can modify the structural and functional development of the remaining modalities and recruit these to drive the deprived cortical areas. In the human visual system, perceptual tasks, electrophysiological and neuroimaging experiments have principally concentrated on congenital or neonatal blind subjects to study this cross-modal type of plasticity (Sadato et al., 1996, 2002; Cohen et al., 1997; Hamilton and Pascual-Leone, 1998; Lessard et al., 1998; Pascual-Leone et al., 2005; Ptito et al., 2005; Collignon et al., 2013; Lazzouni and Lepore, 2014).

### Lessons from Blind Mammals

In early blind (binocular enucleated) mammals, territories associated with somatosensory and auditory functions appear expanded and recruit the former visual areas, including V1, to perform increased multimodal processing (Rauschecker et al., 1992; Toldi et al., 1994a; Izraeli et al., 2002; Laemle et al., 2006; Laramée et al., 2011; Charbonneau et al., 2012).

At the systems level, one possible mechanism is the rewiring of long-range subcortical connectivity patterns (Karlen et al., 2006). Indeed, the inferior colliculus (IC), a midbrain auditory nucleus that normally projects to the primary auditory thalamic nucleus (medial geniculate nucleus, MGN) in sighted animals, can additionally connect with the dorsal LGN and thereby convey non-visual information to V1 in experimentally blind animals (Piché et al., 2004, 2007; Chabot et al., 2007, 2008) as observed in the naturally blind mole rat *Spalax ehrenbergi* (Bronchti et al., 2002). Similarly, somatosensory afferents can form an alternative route to innervate the LGN in order to transfer tactile inputs to regions normally devoted to visual processing (Asanuma and Stanfield, 1990). In addition, the higher-order lateral posterior thalamic nucleus can also constitute to an anatomical pathway for the transmission of somatosensory-driven responses to the rat visual cortex upon neonatal binocular enucleation (Négyessy et al., 2000).

In the mature brain, severe deprivation will also cause a time-dependent cascade of reorganization across allocated neural networks. Preexisting connections carrying information of the other senses are rapidly unmasked and strengthened, leading to long-term structural adjustments including new synapses. Although the examples of subcortical involvement are mostly present following early deprivation, it is proposed that hearing impairment in adult ferrets results in cross-modal cortical reorganization originating from alterations in the brainstem, which in normal animals already receives multimodal inputs (Shore et al., 2008; Allman et al., 2009b). In support of the latter, hearing loss also enhances somatosensory innervation of the dorsal cochlear nucleus (auditory brainstem) (Shore et al., 2008).

Other system-level hypotheses have been described that could prevail in both juvenile and adult blind subjects. The first implies changes in cortico-cortical feedback, in which existing projections from multimodal higher-order cortices (i.e., frontal, parietal and temporal association cortex) increase their influence onto primary sensory cortices (Newton et al., 2002; Lippert et al., 2013; Lingnau et al., 2014). Additionally, changes in direct and indirect cortico-cortical connections, between different primary sensory cortices can account for a cross-modal aspect of plasticity, at least at the functional level (Wang et al., 2012; Sieben et al., 2013). Multimodal neurons in primary cortices that work as information hubs that regulate multisensory cortical recruitment under different conditions of sensory stimulation or deprivation, could add to these existing connections modulating cross-modal changes (Vasconcelos et al., 2011). Apart from tracer studies in V1 of adult opossums enucleated at birth (Karlen et al., 2006), evidence for large-scale structural changes of cortical afferents to V1 is lacking in mouse models of congenital or neonatal blindness (Laramée et al., 2011, 2013; Charbonneau et al., 2012; Wang et al., 2012; Sieben et al., 2013). This is in agreement with intermodal connections between primary sensory cortices whose presence was already shown in intact rodents (Larsen et al., 2009; Campi et al., 2010; Iurilli et al., 2012; Stehberg et al., 2014; Henschke et al., 2015) and suggests that their development is mainly unaffected by early blindness. Recently, cross-modal potentiation of thalamocortical axons in non-deprived primary sensory cortices of the mouse is put forward as a general mechanism of adult synaptic plasticity in response to short sensory deprivation (Petrus et al., 2014). Dark exposure and cochlear inactivation were used as sensory deprivations in adult mice. Functional changes in the non-deprived primary cortex were evaluated using *in vivo* single unit recordings to characterize tuning properties and *in vitro* optogenetic activation of thalamocortical axons combined with mEPSCs in layer IV neurons to dissect alterations in synaptic transmission. Dark exposure altered the tuning properties of auditory neurons and increased the synaptic responsiveness in layer IV neurons of A1 upon optogenetic activation of MGB neurons. However, it did not affect the strength of V1 layer IV inputs originating from geniculate neurons since the critical period for geniculocortical axon plasticity within the visual system had already passed. Deafening induced specific potentiation of geniculocortical inputs of the visual cortex without affecting granular neurons in A1. Together, these findings suggest that deprivation of one sensory input results in the subsequent strengthening of thalamocortical projections in the non-deprived primary cortices in adulthood.

What kind of molecular mechanisms mediate this experience-dependent plasticity and which neural networks are susceptible to cross-modal reorganization at any given age remains largely unknown. These queries deserve attention because they are essential for understanding the specific development of each sensory system and their multimodal interactions (Bavelier and Neville, 2002). At the molecular level, cross-modal homeostatic plasticity in the primary sensory cortices of juvenile mice (P28) has been associated with changes in AMPA receptor subunits. Excitatory postsynaptic transmission was scaled up in V1 upon dark exposure while opposite changes in mEPSC amplitudes and AMPA receptor 1 expression, phosphorylation and rectifying properties were discerned in S1 (Goel et al., 2006). The latter likely reflect a homeostatic response to increased activity of the spared senses upon transient blindness. In addition, synapse-specific strengthening or LTP of layer IV to layer II/III inputs in the barrel cortex of juvenile rats occurs after visual deprivation (dark exposure for 2 days) and is mediated by serotonin-signaling-dependent delivery of the AMPA receptor 1 subunit to the synapse. These cross-modal alterations ultimately sharpen the tuning of barrel neurons in response to principal whisker stimulation (Jitsuki et al., 2011). Epigenetic changes, namely H4 deacetylation, are additional mechanisms that orchestrate the expansion of the barrel cortex following binocular enucleation in rats (Fetter-Pruneda et al., 2013). Together, these advances have led to subsequent whole cell recording experiments in juvenile mice with a different degree of visual deprivation (dark exposure, binocular enucleation and bilateral lid suturing) that confirmed distinct, independent functions and sensory requirements of unimodal vs. cross-modal synaptic plasticity. Complete loss of vision is necessary to induce unimodal scaling whereas loss of patterned vision is sufficient to induce cross-modal alterations in synaptic scaling (He et al., 2012).

### Monocular Enucleation can also Induce Cross-Modal Reorganization

Given that the timing or age of vision loss, in addition to the degree (complete or partial) and type (natural or experimental) of deprivation, has a strong influence on the nature of cross-modal plasticity, the effects of ME during development, adolescence and adulthood will be discussed separately.

#### Early Monocular Enucleation (At Birth, P0)

Apart from unimodal changes in visually evoked response maps, in neonatal rats ME triggers cross-modal changes including the invasion of somatosensory cortex in the contralateral visual cortex (V1 and V2) since bimodal neurons are more frequently found within these visual areas (Toldi et al., 1988, 1994a,b). Multisensory interaction experiments confirmed that somatosensory-evoked potentials are generated within the visual cortex and are not passively conducted from the somatosensory cortex (Toldi et al., 1988). In contrast, auditory activation maintains its territory as in normally sighted rats and does not invade the visual cortex upon ME at birth (Toldi et al., 1988). Massive cross-modal plasticity was further explored by combining the electrophysiological and autoradiographic detection of tactile responses in the visual cortex evoked by both electrical and mechanical whisker stimulation. A widespread expansion of the somatosensory responsive area is observed along the antero-posterior axis (Toldi et al., 1988, 1994b), indicating that neonatal ME also exerts a strong influence on the somatosensory system itself. These cross-modal interactions will likely provide the neural basis for behavioral compensation(s).

#### Effects of Monocular Enucleation in Adulthood (Mouse, P120)

So far, only few studies could prove that cross-modal changes are also manifested at the cortical level in partially deprived adults. Indeed, functional reorganization by unmasking of mature but silent intermodal connections in adult monocularly enucleated rabbits (P140) has been demonstrated (Newton et al., 2002). Moreover, in adult ferrets (P189–240) with moderate hearing loss new multisensory neurons, yet a few that show multisensory integration, are detected in the deprived core auditory cortex (Meredith et al., 2012). However, their functional meaning and consequences to behavior remain largely unclear and could even be responsible for maladaptive perceptual effects such as tinnitus (Allman et al., 2009a; Meredith et al., 2012).

**Figure 1:**
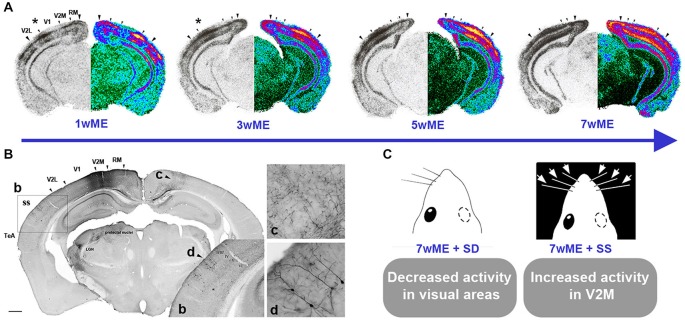
**Spatiotemporal reactivation of the contralateral visual cortex by visual and cross-modal inputs after monocular enucleation (ME) in adult mice. (A)** Layer- and time-specific recovery of neuronal activity in the left visual cortex subsequent to removal of the right eye at an adult age of P120 is illustrated. Molecular activity profiles of the visual cortex have been assessed by the *zif268* mRNA expression analysis around Bregma level −3.40 mm. For each section, the original autoradiogram displaying the deprived (left) visual cortex is shown in gray and its matching pseudo-colored mirror image. The medial and lateral extent of the left visual cortex is marked by the two large arrowheads whereas small arrowheads delineate the interareal boundaries. The activity in the central binocular cortex starts to expand supragranularly (asterisk first and second panel) between 1 and 3 weeks post-ME. Between 3 and 5 weeks, infragranular layers also start to show increased reactivation. **(B)** Anterograde and retrograde transport of fluororuby upon injection in V2M of a 7wME mouse. b: Detail of Fluororuby signal at the location of somatosensory cortex: tracer is transported in an anterograde way to axon terminals in layers V and VI, while supragranular layers II/III contain retrogradely labeled cell bodies and dendrites. c: Detail of anterogradely labeled fibers in contralateral V2M. d: Detail of retrogradely labeled pyramidal cells in layers II and III of ipsilateral/adjacent somatosensory cortex. **(C)** Subsequent whisker manipulations in 7wME mice were employed in order to verify the functional relevance of the intermodal connections in the ME-induced reactivation profile. Somatosensory deprivation (SD) by trimming the right-side vibrissae results in decreased visual cortex activity whereas somatosensory stimulation (SS) through exposure to toys and novel objects in the dark increased activity, especially in V2M. Adapted from Van Brussel et al. (2011).

In line with these studies, Van Brussel et al. (2011) discerned a partial non-visual contribution to the restoration of cortical activity in the visual cortex of adult mice (P120) following long-term ME (7 weeks) (Figure 1). Establishing expression maps for the activity reporter gene *zif268* allowed comparison of neuronal activity between the monocular and binocular contralateral visual cortex of control and ME mice. ME first induced the potentiation of ipsilateral open-eye input leading to the reactivation and expansion of the binocular territory. Next, a slower unmasking of preexisting long-range cross-modal projections occurred, facilitating the transfer of tactile information to the extrastriate cortex. This interpretation was further substantiated at the functional level by ipsilateral whisker deprivation as well as whisker stimulation experiments that respectively reduced and increased visual cortex activity, especially in the medial monocular cortex of long-term ME mice (Figure 1C; Van Brussel et al., 2011). Indeed, in control mice such whisker manipulations only influence molecular activity in the barrel cortex and not in the visual cortex. The lack of changes in molecular activity in the visual cortex of control mice combined with the clear effect in adult ME mice put the conversion of silent or subthreshold multimodal input into suprathreshold input forward as a substrate of the ME induced cross-modal plasticity (Van Brussel et al., 2011).

A high incidence of multisensory neurons has been detected in so-called “transition zones” between the primary areas of different modalities (Toldi et al., 1986; Wallace et al., 2004). In light of these multisensory zones, it was not surprising that the multimodal reactivation of molecular activity in adult mice appeared starting from the anterior and lateral borders of visual cortex with somatosensory and auditory cortex.

#### Monocular Enucleation in Adolescence (Mouse P28–P60)

In mouse and rat, eye opening occurs around the age of 12–14 days (P12–14). It is assumed that puberty starts around P28 and adolescence lasts until approximately the 56th postnatal day. This transition period between P28 and adulthood (P120) corresponds to the physiological age window of human adolescence (Han et al., 2005; Brenhouse and Andersen, 2011). Despite massive synaptic rearrangements, functional changes and gene expression modifications upon ME (Majdan and Shatz, 2006), this age interval is often overlooked in typical visual plasticity research, including ocular dominance plasticity (but see Daw et al., 1992; Majdan and Shatz, 2006; Lehmann and Löwel, 2008; Huang et al., 2010). Important structural alterations occur in the adolescent brain, including the visual cortex, and this without a large effect on overall volume. For example, all layers in V1 of the macaque monkey undergo intensive synaptogenesis during early postnatal life, followed by a slow decrease in synaptic density in the next years (Bourgeois and Rakic, 1993). Thereafter, a rapid reduction of excitatory synapses situated on dendritic spines is observed around the age of puberty. This period of synaptic pruning seems to manifest itself more quickly in layer IV compared with supra- and infragranular layers (Bourgeois and Rakic, 1993). Likewise, certain perceptual abilities (i.e., contour integration) related to the ventral stream of the human visual system do not develop until well into adolescence (Kovács, 2000), and it is likely that a gradual maturation of particular molecular and cellular characteristics largely support these late aspects of visual cortex functioning (Bourne and Rosa, 2006).

When ME is applied to adolescent mice (P45) an incomplete reactivation of the deprived visual cortex was detected, even after 7 weeks, due to the lack of a clear take-over of the visual cortex by somatosensation (Nys et al., 2014). This was a quite surprising observation since different types of plasticity, including ocular dominance plasticity, are often more elaborate in young animals compared to older ones. The current identification of a pre-adult period for cross-modal plasticity resulting from ME is quite remarkable but may not be generalized to cross-modal plasticity following other deprivation paradigms. There are undoubtedly multiple critical periods for different types of plasticity across different subdivisions of the visual cortex, and in fact this is what we want to bring forward with this review.

## Consequences of ME on the Human Visual System

Most of the studies of plasticity in humans have focused on the effect of ME during early development whereas studies on late ME subjects are rather limited. From a medical perspective, negative consequences of ME have been described in adult patients where unilateral enucleation can cause complex visual hallucinations even if they have excellent vision in the remaining eye (Ross and Rahman, 2004). Phantom pain or sensation is also frequently encountered after eye amputation (i.e., ME) in adult patients, whereas it is rather unlikely in children (Flor et al., 2006; Rasmussen et al., 2011).

### Effects of Early ME on the Human Visual System

Just like in ME animals, the loss of stereoscopic vision has been studied in unilateral enucleation patients (for review see Steeves et al., 2008). Upon early ME in humans, the location of the visual egocenter is altered due to a shift towards the open ipsilateral eye, inducing an asymmetric bias. However, after a prolonged period with one-eyed vision, the spatial processing system recalibrates to adapt to their new monocular world. Hence, the egocenter is restored to an anatomically symmetrical location (Hoover et al., 2012). In relation to intramodal changes of visual processing upon early enucleation, some features of ventral stream functions, such as visual spatial (contrast-defined visual) abilities, are enhanced in enucleated individuals compared with monocular viewing controls or are at least equal to binocular viewing controls (Nicholas et al., 1996; González et al., 2007). In contrast, visuo-spatial memory (Cattaneo et al., 2008) and dorsal stream functions such as motion perception, oculomotor behavior and speed perception are negatively affected by early ME since these functions strongly rely on normal binocular experience early in life (Steeves et al., 2002; Burnat et al., 2012; Kelly et al., 2013; Zapasnik and Burnat, 2013; González et al., 2014). A strong age-at-enucleation effect is present since it determines the amount of behavioral compensation achieved during monocular vision (Marotta et al., 1995; Nicholas et al., 1996). In addition, morphological changes in subcortical structures such as the optic nerve, optic chiasm, optic tract and LGN, reported in early enucleated subjects differ from the ones detected after late enucleation (Horton and Hocking, 1997; Kelly et al., 2013). Although, improvements in low- to mid-level spatial abilities are observed, early ME seems to impair development of higher-spatial functions such as face perception in one-eyed humans (Kelly et al., 2012). Other visual processing parameters such as horizontal saccade dynamics are unchanged in monocular viewing people compared with those with normal binocularity suggesting that the afferent (sensory) and efferent (motor) pathways from the saccadic system are not functionally impaired (González et al., 2013).

Aside from the unimodal, within-visual system effects, the complete loss of one eye additionally recruits cross-modal adjustments in the auditory system that support improved sound localization in monocular blind subjects (Hoover et al., 2012). Many perceptual skills do not merely rely on one sense but are established via the integration of different congruent sensory stimuli (visual, tactile, auditory or olfactory) to maximally extract information from the environment (Meredith, 2002). In additional behavioral experiments, people with one eye show no Colavita effect (visual dominance and auditory ignorance in a bimodal stimulation task) but instead reveal equal preference for visual and auditory stimuli (Moro and Steeves, 2012). When Moro and Steeves (2013) adapted the stimulation protocol of the Colavita task in favor of audition by increasing the temporal processing (repetitive stimuli), the expected reverse Colavita effect was absent in one-eyed people (Moro and Steeves, 2013). Accordingly, the enhanced auditory localization capacity following early ME is not sufficient to allow an auditory dominance in the temporal version of the Colavita task suggesting impartial multisensory processing.

## Neural Mechanism of Cross-Modal Plasticity: Importance of Multimodal Regions and Relation to ME-Induced Plasticity

### Observations in the Blind: Cortical Function Specificity and Age-Effects

Multisensory integration is shown to occur widely along the neuroaxis, including primary sensory areas which are often regarded as unisensory (Shimojo and Shams, 2001; Wallace et al., 2004; Cappe and Barone, 2005; Kayser and Logothetis, 2007; Vasconcelos et al., 2011; Henschke et al., 2015) and has an essential role in the following “supramodal” skills: spatial localization, shape recognition and motion detection. Multimodal associative areas (Lingnau et al., 2014) and to a lesser extent homologous neuronal populations in early cortical areas that subserve these “supramodal” abilities are exactly the ones that mediate cross-modal reorganization and enhanced performances of intact modalities after the loss of a sense. It thus seems that cross-modal plasticity is not a global phenomenon but rather induces specific changes in functional abilities while leaving others unaltered. In other words, specific circuits of the deprived visual cortex in the early blind will use their repertoire of computational properties (laid down by early development and genetics) to perform similar functions for audition, only now they have a different input source (Oliveira-Silva et al., 2007; Bavelier and Hirshorn, 2010; Renier et al., 2014). Specifically deactivating the functionally homologous regions of the deprived cortex by transmagnetic stimulation (Cohen et al., 1997; Vargas et al., 2001; Wang et al., 2005; Merabet and Pascual-Leone, 2010) or cryogenic cooling in cats (Nakamura et al., 1984; Riback and Robertson, 1986; Lomber et al., 2010) abolishes the better performance in multimodal skills using the remaining senses. These manipulations corroborate the hypothesis that the behavioral function of cross-modal plasticity in a specific area is related to its role in normally hearing/sighted individuals as recently shown by single unit recordings in cats (Chow et al., 2011; Meredith et al., 2011) and by functional magnetic resonance imaging in humans (Valverde, 1968; Heumann and Rabinowicz, 1982; Renier et al., 2010; Lingnau et al., 2014).

Considering the theory of “preserved function”, it should be noted that the preservation of visual perceptual properties could guide cross-modal plasticity presumably if vision is lost early in life (Illig et al., 1998; Collignon et al., 2013). In agreement with this finding, some studies indicate a critical period for cross-modal plasticity in the blind based on the performance in non-visual perception and cognitive tasks (Buchel et al., 1998; Cohen et al., 1999; Sadato et al., 2002; Steeves et al., 2008). For example, mathematical modeling estimated that auditory activation of V1 in congenital blind subjects is mediated by direct functional connections between A1 and V1 whereas auditory-driven activity in V1 from late blind subjects is largely derived from feedback projections of the parietal cortex (Collignon et al., 2013).

Only a few extrastriate areas (bilateral cuneus) involved in depth perception were significantly more activated in congenital compared with the late blind suggesting also region-specific cross-modal plasticity (Avwenagha et al., 2006; Collignon et al., 2013). Complementary to the extensive functional and behavioral studies in blind subjects, a recent study addressed the age-dependent structural and topological modifications in cortical networks to determine at which age the brain network properties are affected by visual deprivation (Li et al., 2013). Following the comparison of four age groups, namely congenital, early, adolescent and adult blind human subjects, it was shown that early blindness decreases global network efficiency while late-onset blindness was characterized by a diminished local efficiency. The largest differences compared with sighted controls were found after congenital blindness and the smallest between adolescent and late blind subjects. The authors conclude that the overall differences in structural alterations mirror the complexity of neurodevelopment, plasticity and disuse in blind people.

The described enhancement of certain perceptual abilities in the congenital or early blind should not lead to the misconception that blind subjects can compensate everything through increased sensitivity of the remaining senses. Still many aspects of tactile and auditory processing are impaired because early sight loss disrupts cross-sensory calibration during development (Gori et al., 2008, 2010).

### Putative Mechanisms of ME-Induced Plasticity: Outlooks

Table 1 shows an overview of the candidate mechanisms underlying ME-induced reorganization early (around birth) or later in life (around adolescence and adulthood). It is plausible that a cortico-cortical framework is responsible for the observed plasticity later in life, in line with what has been described in blind subjects (Klinge et al., 2010) and well characterized for cortical map reorganization (Darian-Smith and Gilbert, 1994, 1995) after less extensive partial deprivations.^3^ The amount of anatomical input does not always correctly reflect the strength and significance of cortical pathways. Together with studies examining the synaptic properties and functional activation of cortical networks, a more accurate characterization of the cortico-cortical connections has been established in mouse. This has been done within a modality (i.e., V1 and V2 (De Pasquale and Sherman, 2011; Ko et al., 2011); A1 and A2v (Covic and Sherman, 2011)) as well as between numerous intra- and interhemispheric cortical areas using the stimulation of arbitrary neuronal populations by optogenetics combined with voltage-sensitive dye imaging as a high resolution readout (Lim et al., 2012). In the context of putative alterations in hierarchical cortical processing within and between senses, information from lower cortical areas (i.e., primary cortex) is not only transferred directly to higher-order cortical areas via cortico-cortical connections (Felleman and Van Essen, 1991) but also indirectly through cortico-thalamo-cortical projections (for review see Guillery and Sherman, 2002; Sherman and Guillery, 2011). Indeed, a tracer study of Négyessy et al. (2000) uncovered cross-modal plasticity across a cortico-thalamo-cortical pathway, which transmits somatosensory information from the barrel cortex via the LP nucleus to V1 in enucleated rats (Négyessy et al., 2000).

**Table 1:** **Candidate mechanisms at different levels underlying visual (U) and cross-modal (CM) plasticity following early and late-onset ME and in comparison with binocular enucleation (BE) or dark exposure (DE) effects**.

Level	plasticity mechanism	uni (U)/cross-modal (CM)	early ME or visual deprivation (neonatal or short after birth)	late-onset ME or visual deprivation	reference
system	Reorientation of retinogeniculate or retinotectal axons from open eye	U	√	/	Toldi et al. (1996)
system	Sprouting of retinogeniculate or retinotectal axons from open eye	U	√	/	Toldi et al. (1996), Chandrasekaran et al. (2005)
system	Respecification of representations in subcortical nuclei	U + CM	√	?	Lund et al. (1973), Toldi et al. (1988, 1994b)
system	Sprouting of thalamocortical axons	U	√	+/−; DE	Antonini et al. (1999), Montey and Quinlan (2011), Yu et al. (2012)
system	Proliferation of cortico-cortical connections	U + CM	√	+/−	Valverde (1968), Laramée et al. (2011)
system	Rearrangements of cortico-cortical connections	U + CM	√	√	Van Brussel et al. (2011), Vasconcelos et al. (2011)
system	Rearrangements of callosaII connections	U + CM	√	/	Laing et al. (2014), Olavarria et al. (2012)
system	Rearrangements of cortico-thalamo-cortical connections	U + CM	ME: ?, BE: √	?	Négyessy et al. (2000), Sherman and Guillery (2011)
system	Modulation of oscillatory activity patterns	U + CM	ME: ?, BE: √	?	Schepers et al. (2012)
synaptic	Changes in multisensory integration	U + CM	√	√	Dehner et al. (2004), Iurilli et al. (2012)
synaptic	Formation of new synapses; spines and axon boutons	U + CM	√	√	Valverde (1968), Kossut (1998)
synaptic/molecular	Strenghtening of synapses/ connections				
	*Plasticity of thalamocortical axons*	U	?	DE	Goel et al. (2006), Jitsuki et al. (2011), He et al. (2012)
	*Plasticity of thalamocortical axons*	CM	√	√	Yu et al. (2012), Petrus et al. (2014)
synaptic	Unmasking of silent existing synapses	U + CM	√	√	Newton et al. (2002), Van Brussel et al. (2011)
molecular	Changes in neurotransmitter release	U + CM	√	√	Nakamura et al. (1984), Riback and Robertson (1986)
molecular	Epigenetic chromatin remodeling	U + CM	ME: ?, BE: √	?	Fetter-Pruneda et al. (2013)
molecular	Shift in the excitation- inhibition balance	U + CM	√	√	Desgent et al. (2010), Ni et al. (2010), Zhang et al. (2013)

On top of cortico-cortical connections and transthalamic loops, a recent study by Petrus et al. (2014) puts cross-modal potentiation of thalamocortical synapses in the non-deprived primary cortices forward as a general mechanism of functional adaptation in the adult cortex upon a short loss of one sensory input. The combined and balanced alterations in thalamocortical and intracortical circuits may support both enhanced feed-forward processing along the non-deprived senses and efficient unmasking of multimodal connections in the sensory deprived areas (Toldi et al., 1986; Wallace et al., 2004; Yu et al., 2013).

Despite lack of direct evidence, it is very likely that structural plasticity is part of the response to ME. In the somatosensory cortex of adult mice, long-term (8 weeks) vibrissectomy (whisker trimming but in this case with one row intact) increases the spine density on basal dendrites of layer V pyramidal neurons and induces the elongation and higher level of branching of axons resident in the spared barrel column compared with the deprived column (Kossut, 1998). Given the 7 weeks time course of the adult ME-induced reactivation (see Section Effects of monocular enucleation in adulthood (mouse, P120); Figure 1), it is also likely that after unmasking and strengthening of open-eye and multimodal inputs, sprouting of cortico-cortical afferents is a structural mediator of the visual and cross-modal reorganization (Table 1). The tracer study in Van Brussel et al. (2011) revealed no large-scale differences in connectivity patterns of V2M in long-term enucleated adult mice or control animals, which is in agreement with the findings of Charbonneau et al. (2012) in intact adult mice. However, it is conceivable that ME-specific structural changes are present but can only be uncovered in a more detailed analysis of axon boutons after anterograde tracer injections or high-resolution fluorescent microscopy in transgenic mice and analysis of spine density and dendritic morphology for instance by means of Golgi-cox impregnation (Aerts et al., 2014b).

These reports and future work with respect to connections will not only expand our knowledge regarding age-dependent and laminar-specific mechanisms of plasticity but will also contribute to a better understanding of mouse (Paperna and Malach, 1991; Zingg et al., 2014) and rat (Paperna and Malach, 1991) cortical networks, including the inhibitory microcircuitry (Pfeffer et al., 2013), and the whole-brain connectome (Oh et al., 2014; Sporns and Bullmore, 2014). In light of these efforts about the structural and functional characterization of intramodal, intermodal and callosal connections, studies that will link the stability and plasticity of these connections with specific behavior and experiences will definitely accelerate future discoveries in the healthy and the diseased brain.

The variation in adult (see Section Effects of monocular enucleation in adulthood (mouse, P120)) and pre-adult plasticity (see Section Monocular enucleation in adolescence (mouse P28-P60)) in reaction to ME in the mouse (Van Brussel et al., 2011; Nys et al., 2014) can in part be explained by the fact that the networks of neurons, neurotransmitter systems (Herlenius and Lagercrantz, 2004), gene regulation patterns as well as their extracellular environment may still change over time (Berardi et al., 2003; Karmarkar and Dan, 2006; Putignano et al., 2007). The GABAergic network, on one side of the excitation-inhibition balance, is a well-know factor in controlling the age-dependent expression of diverse types of plasticity (Hensch, 2003; Keck et al., 2011). It tightly regulates the activity of cortico-(thalamo-)cortical inputs (Callaway, 2004) and it has been suggested to be involved in multisensory integration (Meredith, 2002; Friedel and van Hemmen, 2008; Olcese et al., 2013). However, the limited knowledge regarding the organization of GABA microcircuits across sensory cortices hampers the constructive prediction of cross-modal changes between inhibitory neurons or between inhibitory and excitatory neurons in the mouse. Two hypotheses were described so far. First, studies in the ferret (Heumann and Rabinowicz, 1982; Pallas, 2001) and hamster (Desgent et al., 2005; Farley et al., 2007) indicated a non-stereotypical but modality-specific organization in the primary sensory areas, A1 and V1, suggesting that early cross-modal plasticity may require experience-dependent adjustments in the number and distribution of specific interneuron subtypes to shape the receptive fields of newly acquired inputs (Desgent et al., 2010). Alternatively, they may adopt a new GABAergic configuration to control or amplify oscillatory activity carrying multisensory information (Lakatos et al., 2007). A second study by Clemo et al. (2003) in cat anterior ectosylvian cortices on the other hand revealed a similar distribution of GABAergic markers across higher-order cortices representing different modalities, suggesting a canonical circuit for sensory processing (Clemo et al., 2003). The different results found in the two studies can be traced back to differences in species, cortical areas and hierarchical level and subset of GABA-related proteins investigated. The presence of multiple types of molecularly and functionally divergent inhibitory interneurons (for a review see Markram et al., 2004) also imposes another degree of variation and complexity in this matter.

Slow working pharmacological manipulations could test the hypothesis that shifting the excitation-inhibition balance supports the ME-induced reorganization in mouse visual cortex. Diazepam (Hensch et al., 1998) or muscimol (Caleo et al., 2007) have often been used as GABA_A_ receptor agonists to increase inhibitory function, whereas picrotoxin (PTX, a GABA_A_ receptor antagonist) and mercaptopropionic acid (MPA, an inhibitor of GABA synthesis) (Harauzov et al., 2010) decrease inhibition. More refined techniques such as muscimol-releasing Elvax implants can furthermore reveal the cortical (sub)regions involved and the physiological and behavioral underpinnings of age-specific reactivation profiles (Smith et al., 2004). These agents are interesting since some features of ME-induced cortical plasticity indeed occur on a longer time-scale. However, the presence of multiple types of inhibitory interneurons as well as the fast millisecond time scales which neurons communicate with, limit the investigative power of these pharmacological receptor agonists. A more sensitive neuromanipulation technique such as optogenetics, a technique incorporating light-inducible channel proteins into specific neuronal cell types, is showing great promise to causally investigate cell type specific functions in awake, behaving animals, offering the possibility to combine this approach with electrophysiological or behavioral readouts to acquire functional information (Yizhar et al., 2011).

## Contribution of the ME Model to the Clinical Relevance of Cortical Plasticity

From a clinical perspective, the relevance of fundamental research using vision impairment models is reflected in the high prevalence of vision-impaired and blind patients worldwide. 285 million people globally suffer from some form of vision deficiency, of which 8% are blind (Pascolini and Mariotti, 2012). Notwithstanding the fact that many eye-diseases can be treated at eye level, it is becoming increasingly evident that frequently occurring vision impairments such as glaucoma can be caused by or are associated with cortical changes (Baroncelli et al., 2011). In this regard, ME studies increase our understanding of how the brain copes with altered vision and age-dependent cortical deficits or functional alterations underlying distinct visual disorders. ME also replicates unilateral vision loss of human patients following ophthalmic trauma, inflammation, injury or enucleation as a common treatment for end-stage glaucoma, retinoblastoma or *Phthisis bulbi* (a shrunken, non-functional eye) (Moshfeghi et al., 2000; Setlur et al., 2010).

Being able to modulate plasticity in a certain direction could attain the best functional and behavioral outcome in a given patient or situation. It will offer great promises in the quest for new therapeutic strategies for neurological disorders or brain injuries although in general, caution is warranted for interventions that tap into brain structure and function to enhance or lower plasticity. At any given time point, an optimal balance between plasticity and stability must be retained.

In this context, the mouse has emerged as the model of choice as it offers unique advantages including molecular and genetic tools to monitor, label and manipulate specific neuronal subtypes or circuits (Huberman and Niell, 2011). Furthermore, great strides have been made in supporting the idea that plasticity mechanisms in mammals can be studied in mice and that its sensory systems are more complex than originally believed.

In relation to ocular dominance shifts, adult visual cortex plasticity following MD can be elevated by impinging on the cortical excitation-inhibition balance and molecular or structural brakes that were established and maintained in a use-dependent manner during postnatal development (Di Cristo, 2007; Hensch and Bilimoria, 2012). Indeed, pharmacological (Pizzorusso et al., 2002; Maya-Vetencourt et al., 2008; Harauzov et al., 2010), genetic (Hensch et al., 1998; Fagiolini and Hensch, 2000; Syken et al., 2006; Carulli et al., 2010) and housing (He et al., 2006; Sale et al., 2007; Huang et al., 2010; Tognini et al., 2012) interventions revealed the possibility to rapidly restore ocular dominance plasticity in adult rodents by circumventing the inhibitory and extracellular matrix limitations on binocular visual cortex plasticity. This explosion of invasive and noninvasive interventions that induce adult ocular dominance plasticity or restore visual acuity in adulthood have moved the field of visual plasticity research and clinical interventions for amblyopia forward apace. As a result, the combination of targeted pharmacological (invasive) manipulation, action video game training to enhance neuromodulation (non-invasive) (Green et al., 2010) and even brain stimulation (Fregni and Pascual-Leone, 2007) to directly tap into the excitation-inhibition balance could reinstate a sensitive period and improve low-level as well as high-level vision in the weak eye. This in turn could guide the treatment of cortical deficits that accompany amblyopia developed early in life, and glaucoma, cataract or macular degeneration often manifested later in life (Dekeyster et al., 2015). Several of the molecular principles governing plasticity outcome in amblyopia are conserved and have been found to occur in the injured brain (Imbrosci and Mittmann, 2011). Insights in age-dependent plasticity gathered by both animal and human research also opens the door to develop new strategies for enhanced learning and memory, for the treatment of mental illness, and for functional rehabilitation following cortical injuries **(**stroke, ischemia or trauma). When damage to the brain occurs due to cancer, stroke or lesions, post-operative or post-lesion training (non-invasive) of sensorimotor and cognitive functions can enable recovery-based plasticity to improve the quality of life for the patient.

With regard to cross-modally driven plasticity, the larger proportion of intrinsic multimodal connections found in the lissencephalic rat and mouse neocortex, even in primary areas, (Paperna and Malach, 1991; Budinger et al., 2006; Wang et al., 2012; Olcese et al., 2013; Hishida et al., 2014) compared to the cat, ferret (Meredith et al., 2012) or monkey, has two important consequences. First, it is plausible to observe ample cross-modal reorganization along the antero-posterior and medio-lateral extent of the visual cortex after visual input loss, especially in higher extrastriate visual areas. Second, widespread cross-modal changes in rodents are likely mediated by the unmasking or signal amplification of latent or subthreshold multisensory circuits that were already tuned by multimodal experiences before deprivation (human: Lee et al., 2007; mouse: Olcese et al., 2013). Accordingly, also upon ME the age of deprivation and the location within the visual cortex will determine the relative expression of certain plasticity mechanisms at the systems, synaptic and molecular level that may partially overlap with those active following binocular enucleation or blindness (Karlen et al., 2006; Qin and Yu, 2013; see Table 1). Many studies have focused on the effects of early loss of sensory input while in relation to the human population; partial sensory deprivation in terms of progressive hearing and vision loss and corresponding cross-modal changes are frequently encountered later in life. Therefore, studying adult cross-modal plasticity in the visual (and other sensory) systems is of equal importance.

Although a large focus has been put on the positive aspects of plasticity, it can also be the origin of pathological conditions (Johnston, 2004; Fernandez et al., 2007) or the cause of maladaptation in light of rehabilitation efforts (Sandmann et al., 2012). For instance, cross-modal plasticity is one factor that is responsible for the absence of or reduced success after cochlear implantation,^4^ especially when a long time-interval of deafness was present before the implantation (Harrison et al., 2005). This negative outcome is mediated by the cross-modal functional improvement of non-auditory cortices at the cost of the auditory cortex ability to process electrical stimulation originating from the cochlear device (Lee et al., 2001). Therefore, effective therapy should include the suppression of cross-modal alterations to permit the recruitment of auditory cortex by the new auditory inputs from the implant and to obtain desirable recovery of auditory functions. Likewise, suppression of non-visual processing in the visual cortex of the blind could be required after introducing a retinal implant. Although electrical implants, such as the recently developed Argus IITM epiretinal prosthesis system, may partially restore vision and allow the identification of letters and words (da Cruz et al., 2013), further research is still needed to improve the interpretation of input signals to the visual cortex. It is exactly in that context that plasticity research in laboratory animals will be of great support.

## Conflict of Interest Statement

The authors declare that the research was conducted in the absence of any commercial or financial relationships that could be construed as a potential conflict of interest.

## Footnotes

^1^Sensitive or critical periods are time windows during early life where certain experiences are indispensable and have an instructive role in the central nervous system.

^2^The two genes *zif268* and *c-fos* belong to a family of regulatory or transcription factor-encoding immediate early genes (IEGs) that are considered as accurate activity reporter genes induced without *de novo* protein synthesis in the mammalian (visual) cortex (Chaudhuri et al., 1995; Herdegen and Leah, 1998; Kaczmarek et al., 1999; Arckens et al., 2000; Zangenehpour and Chaudhuri, 2002).

^3^Representing retinal lesions, digit amputations, and trimming of a few whisker rows instead of unilateral removal of an eye, hand or vibrissae along the snout.

^4^Cochlear implant = a microelectrode array implanted in the cochlea that directly stimulates the auditory nerve fibers in hearing impaired subjects.
